# Co-Substitution Effect in Room-Temperature Ferromagnetic Oxide Sr_3.1_Y_0.9_Co_4_O_10.5_

**DOI:** 10.3390/ma13102301

**Published:** 2020-05-16

**Authors:** Akihiro Tsuruta, Shuji Kawasaki, Masashi Mikami, Yoshiaki Kinemuchi, Yoshitake Masuda, Asaya Fujita, Ichiro Terasaki

**Affiliations:** 1 National Institute of Advanced Industrial Science and Technology (AIST), Shimo-Shidami, Moriyama-ku, Nagoya 463-8560, Japan; kawasaki.shuji@e.mbox.nagoya-u.ac.jp (S.K.); m-mikami@aist.go.jp (M.M.); y.kinemuchi@aist.go.jp (Y.K.); masuda-y@aist.go.jp (Y.M.); asaya-fujita@aist.go.jp (A.F.); terra@nagoya-u.jp (I.T.); 2 Department of Physics, Nagoya University, Furo-cho, Chuikusa-ku, Nagoya 464-8602, Japan

**Keywords:** cobalt oxide, elemental substitution, room temperature ferromagnetism, spin state

## Abstract

We investigated the Co substitution effect for the magnetic properties in room-temperature ferromagnetic oxide Sr_3.1_Y_0.9_Co_4_O_10.5_. The substituted element (Al and Ga) and low-spin state Co^3+^, which was changed from a high-spin or intermediate-spin state by Al or Ga substitution, reduced the Curie temperature to even 1.5 times lower than the temperature estimated from a simple dilution effect. Al^3+^ preferentially substituted for intermediate-spin-state Co^3+^ in the ferrimagnetic CoO_6_ layer and deteriorated the saturation magnetization of Sr_3.1_Y_0.9_Co_4_O_10.5_. By contrast, Ga^3+^ substituted for high-spin-state Co^3+^ in the CoO_6_ layer and/or the antiferromagnetic CoO_4.25_ layer and enhanced the saturation magnetization per Co ion. These results indicate that the magnetic properties of Sr_3.1_Y_0.9_Co_4_O_10.5_ can be controlled by selectively substituting for Co^3+^ with different spin states.

## 1. Introduction

Among transition metal oxides with various functions, cobalt oxides are especially interesting compounds and attract attention from many researchers. Layered cobalt oxides (Na_0.5_CoO_2_ and Ca_3_Co_4_O_9_) with unusual thermoelectric properties [1,2,3,4], REBaCo_2_O_5.5_ (RE = rare earth) with giant magnetoresistance [5,6,7], and LaCoO_3_ with temperature-induced spin-state transitions [8,9,10,11,12,13] are typical compounds that have been actively studied. Co^3+^ ions take three types of spin state: the low-spin (LS; *S* = 0) state of (t_2g_)^6^ and the high-spin (HS; *S* = 2) state of (e_g_)^2^(t_2g_)^4^, as well as the intermediate-spin (IS; *S* = 1) state of (e_g_)^1^(t_2g_)^5^. The spin state of Co^3+^ ions is strongly related to the functions and phenomena of the cobalt oxides, and unraveling the origins of these functions has opened up new material design guidelines.

**Figure 1:**
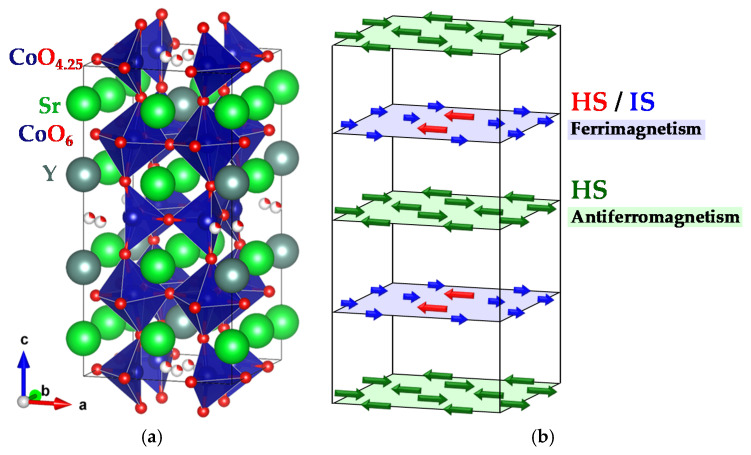
(**a**) Crystal structure and (**b**) magnetic structure of Sr_3.1_Y_0.9_Co_4_O_10.5_.

The A-site-ordered perovskite Sr_3.1_Y_0.9_Co_4_O_10.5_ is a weak-ferromagnetic (ferrimagnetic) material at room temperature. The transition temperature (*T*_c_: Curie temperature) has been reported to be around 340 K for polycrystalline samples [14] and 370 K for single-crystalline samples [15]. The crystal structure of Sr_3.1_Y_0.9_Co_4_O_10.5_ is shown in Figure 1a. The octahedral CoO_6_ layer and the oxygen-deficient (tetrahedral/pyramidal) CoO_4.25_ layer, which consists of tetrahedral CoO_4_ and pyramidal CoO_5_, are stacked alternately [16,17,18,19]. In this system, the two structural-phase transitions have been revealed in Sr_3.12_Er_0.88_Co_4_O_10.5_ [20,21]. The space group of tetragonal *I*4*/mmm* in the highest-temperature phase with a 2*a* × 2*a* × 4*a* unit cell, where *a* is the lattice parameter of the primitive perovskite unit cell, changes into monoclinic *A*2*/m* with a 2√2*a* × 2√2*a* × 4*a* supercell due to oxygen vacancy ordering at 509 K. Further, the lowest-temperature phase, in which the *a*-axis is doubled (4√2*a* × 2√2*a* × 4*a*), appears due to a spin state and/or orbital ordering at 360 K. The origin of ferromagnetism in Sr_3.1_Y_0.9_Co_4_O_10.5_ has been revealed as the ferrimagnetism of the CoO_6_ layer [14,20,22]. In the CoO_6_ layer, both HS- and IS-state Co^3+^ exist, where the majority component is the IS state [23]. All the Co^3+^ ions in the CoO_4.25_ layer take the HS state and align antiparallel to show antiferromagnetic order [21]. Figure 1b shows the magnetic structure of Sr_3.1_Y_0.9_Co_4_O_10.5_. The saturation magnetization simply expected from this structure is 0.25 μ_B_/Co. In this material, a spin-state crossover, which is a change from the HS- and IS-states in the CoO_6_ layer into the LS state, is observed around 150 K. Additionally, it has been reported that the spin-state crossover is enhanced by physical pressure [24] and chemical pressure owing to the pressure-induced enlargement of the crystal-field splitting [25]. In this interesting cobalt oxide, so far, various kinds of investigations [26,27,28,29,30] have been conducted and the A-site substitution has been actively studied in order to reveal and control the spin state of Co ions [25,31,32].

In this study, we substituted nonmagnetic elements of Al and Ga with different ionic radii for the B-site (Co-site) and measured the magnetic properties of Sr_3.1_Y_0.9_Co_4−*x*_*B_x_*O_10.5_ (*B* = Al and Ga: *x* = 0, 0.2, and 0.4). It was expected that the Curie temperature would be reduced towards room temperature by a simple dilution effect. Moreover, the ferrimagnetism of the CoO_6_ layer, which is responsible for the magnetization of Sr_3.1_Y_0.9_Co_4_O_10.5_, was expected to be controlled through selective substitution.

## 2. Experimental

Sr_3.1_Y_0.9_Co_4-*x*_*B_x_*O_10.5_ (*B* = Al and Ga: *x* = 0, 0.2, and 0.4) polycrystalline samples were prepared by a solid-state reaction. SrCO_3_, Y_2_O_3_, Co_3_O_4_, Al_2_O_3_, and Ga_2_O_3_ were mixed and calcined at 1100 °C for 12 h in air. The calcined products were ground, pressed into pellets, and sintered at 1100 °C for 48 h in air. To compensate Co evaporation during calcining and sintering, 5-mol% Co_3_O_4_ was added from the stoichiometric ratio, following the previous report [25].

X-ray diffraction (XRD) patterns of the ground samples were taken with CuKα (*λ* = 1.5418 Å) radiation using a standard diffractometer with monochromator (Rigaku, SmartLab, Tokyo, Japan). The 2*θ*–*θ* scan was carried out with a continuous-scan mode from 20 to 80° at 5°/min. The magnetization was measured from 2 to 350 K by a commercial superconducting quantum interference device magnetometer (Quantum Design, MPMS, San Diego, CA, USA). We applied µ_0_*H* = 0.1 T for the measurement of magnetization–temperature (*M*–*T*) data and measured the magnetization *M* in sweeping field µ_0_*H* from −7 to 7 T at 2 K.

## 3. Results and Discussion

**Figure 2:**
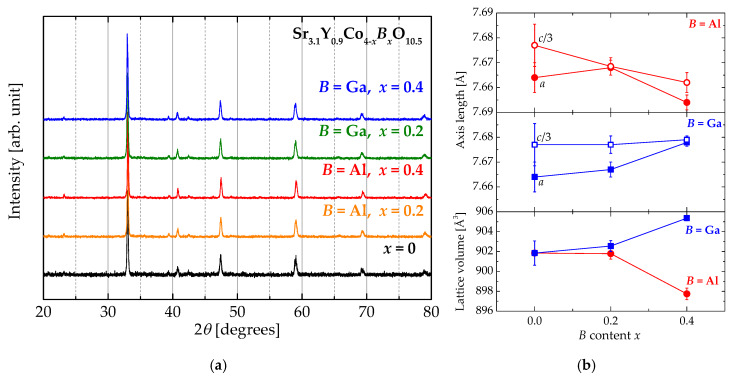
(**a**) XRD (CuKα) patterns of Sr_3.1_Y_0.9_Co_4−*x*_*B_x_*O_10.5_ (*B* = Al and Ga: *x* = 0, 0.2, and 0.4) powders. (**b**) The axis lengths and the lattice volumes of Sr_3.1_Y_0.9_Co_4−*x*_*B_x_*O_10.5_ as a function of *B* content.

Figure 2a shows the XRD patterns of Sr_3.1_Y_0.9_Co_4−*x*_*B_x_*O_10.5_ powders. All the peaks of the samples are indexed as Sr_3.1_Y_0.9_Co_4_O_10.5_ phase without any impurity phases. The 2*θ* values systematically shift according to the substituted element and amount. The axis lengths and the lattice volumes calculated from the XRD patterns are shown in Figure 2b. Both *a*- and *c*-axis lengths of Sr_3.1_Y_0.9_Co_4−*x*_Al*_x_*O_10.5_ are found to decrease with increasing Al content within experimental uncertainties. On the other hand, in Sr_3.1_Y_0.9_Co_4−*x*_Ga*_x_*O_10.5_, the two lengths increase with increasing Ga content. Consequently, the lattice volumes of Sr_3.1_Y_0.9_Co_4−*x*_Al*_x_*O_10.5_ and Sr_3.1_Y_0.9_Co_4−*x*_Ga*_x_*O_10.5_ decrease and increase with increasing *B* content, respectively. The ionic radii of Co^3+^, Al^3+^, and Ga^3+^ are listed in Table 1. In the case of Co^3+^, the radii depend on the spin state. Co^3+^ ions in any spin state are larger than Al^3+^ ions and smaller than Ga^3+^ ions. The changes in lattice volume due to Co substitution are well explained in terms of the ionic radii, but the substitution sites for both Al^3+^ and Ga^3+^ could not be determined from the axis length and the lattice volume.

**Figure 3:**
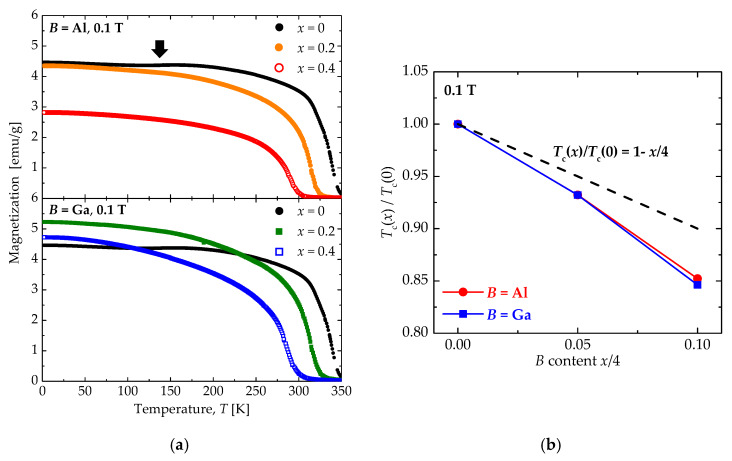
(**a**) Temperature dependence of the magnetization of Sr_3.1_Y_0.9_Co_4−*x*_*B_x_*O_10.5_ (*B* = Al and Ga: *x* = 0, 0.2, and 0.4) under 0.1 T. (**b**) The normalized Curie temperature (*T*_c_(*x*)/*T*_c_(0)) of Sr_3.1_Y_0.9_Co_4−*x*_*B_x_*O_10.5_ by *T*_c_ of Sr_3.1_Y_0.9_Co_4_O_10.5_ as a function of substitution ratio of Co by *B* (*x*/4). *T*_c_ has been estimated from the inflection points in the temperature derivative of the *M*–*T* curve.

Figure 3a shows the field-cooled *M*–*T* curves of Sr_3.1_Y_0.9_Co_4−*x*_*B_x_*O_10.5_. In both cases of *B* = Al and Ga, the magnetic transition shifts to low temperatures with increasing substitution amounts of *B.* The Curie temperature (*T*_c_) is unable to be determined using the Curie–Weiss law because the magnetic transition of this system is of first order. Then, we estimate the *T*_c_ from inflection points in the temperature derivative of the *M*–*T* curves.

The normalized *T*_c_ (*T*_c_(*x*)/*T*_c_(0)) of Sr_3.1_Y_0.9_Co_4-*x*_*B_x_*O_10.5_ by *T*_c_ of Sr_3.1_Y_0.9_Co_4_O_10.5_ is shown in Figure 3b as a function of substitution ratio of Co by *B* (*x*/4). The expected *T*_c_ reduction from a simple dilution effect due to Co substitution is shown by the dashed line. The *T*_c_’s of Sr_3.1_Y_0.9_Co_4−*x*_Al*_x_*O_10.5_ and Sr_3.1_Y_0.9_Co_4−*x*_Ga*_x_*O_10.5_ coincide with each other and are 1.5 times lower than the *T*_c_ expected from the dilution effect. Assuming that the dilution effects is valid, we expect that the Al and Ga substitutions should generate additional nonmagnetic Co ions. In other words, Al or Ga substitution for the Co site may drive neighboring Co^3+^ from the HS or IS state into the nonmagnetic LS state. The magnetization reduction due to the spin-state crossover, which is indicated by the thick arrow in Figure 3a, is observed around 150 K only in Sr_3.1_Y_0.9_Co_4_O_10.5_. Hence, both Al and Ga substitutions suppress the spin-state crossover. The transition from the HS or IS state to the LS state at low temperature is no longer necessary because some Co^3+^ has already been stabilized to the LS state by Al/Ga substitution.

**Figure 4:**
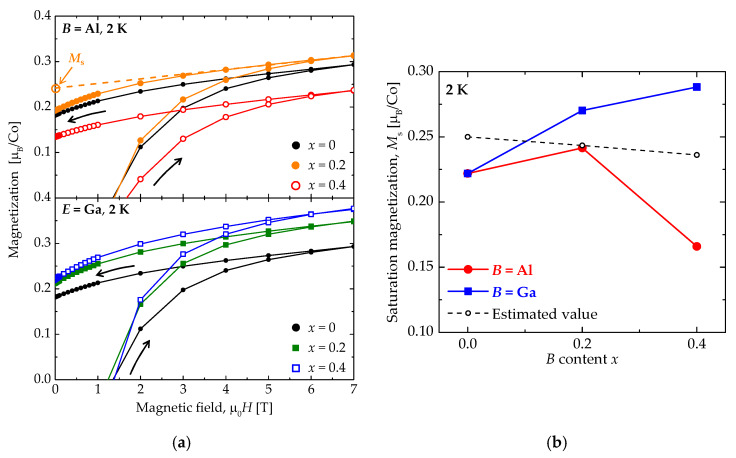
(**a**) Magnetic field dependence of the magnetization of Sr_3.1_Y_0.9_Co_4−*x*_*B_x_*O_10.5_ (*B* = Al and Ga: *x* = 0, 0.2, and 0.4) at 2 K. (**b**) Saturation magnetization *M*_s_ of Sr_3.1_Y_0.9_Co_4−*x*_*B_x_*O_10.5_ as a function of the *E* content *x*.

The magnetic field dependence of magnetization of Sr_3.1_Y_0.9_Co_4−*x*_*B_x_*O_10.5_ at 2 K is shown in Figure 4a. The high-field magnetization depends on the substitution element and amount. Since the Sr_3.1_Y_0.9_Co_4−*x*_*B_x_*O_10.5_ system shows ferrimagnetic order, the magnetization does not saturate but continues to increase with the increasing magnetic field. Then, we define the saturation magnetization (*M*_s_) as the *y* intercept of the linearly extrapolated line from the high-field *M*–*H* curve, as shown by the dashed line in Figure 4a.

The *M*_s_ of Sr_3.1_Y_0.9_Co_4−*x*_*B_x_*O_10.5_ at 2 K is shown in Figure 4b as a function of the *B* content *x*. The *M*_s_ of Sr_3.1_Y_0.9_Co_4_O_10.5_ is smaller than the *M*_s_ = 0.25 μ_B_/Co expected from the magnetic structure because some magnetic Co^3+^ has experienced the spin-state crossover to the nonmagnetic LS state at 2 K. Since *M*_s_ is referred to as the magnetization per Co ion (not per unit cell), *M*_s_ is expected to remain intact if Al or Ga is substituted for Co randomly. If we ascribe the 1.5-times faster reduction of *T*_c_ to nonmagnetic Co ions additionally induced by Al/Ga substitution, Al/Ga substitution drives some portions of the neighboring Co ions to the LS state. We can estimate an amount of LS-state Co ions induced by Al/Ga substitution by assuming that the dilution effect is valid. Taking the estimated amount of LS-state Co ions with Al/Ga random substitution into account, we evaluate *M*_s_ to be 0.243 and 0.236 μ_B_/Co at *x* = 0.2 and 0.4, respectively, as shown by the open circles. The *M*_s_ of Sr_3.1_Y_0.9_Co_4−*x*_Ga*_x_*O_10.5_ is larger than the calculated values and clearly increases with increasing Ga content. On the other hand, the *M*_s_ of Sr_3.1_Y_0.9_Co_4−*x*_Al*_x_*O_10.5_ is smaller than the calculated values and decreases with increasing Al content. These obvious differences between Al and Ga substitutions suggest different substitution sites.

When the HS-state Co^3+^ in the CoO_4.25_ layer is substituted, the magnetization per unit cell will retain the same value because the CoO_4.25_ layer is antiferromagnetic. Note that, since *M*_s_ is referred to as the magnetization per Co ion, the magnetization per unit cell independent of *B* content implies that *M*_s_ increases with decreasing amounts of Co ion in the unit cell due to the substitution. If the HS-state Co^3+^ in the CoO_6_ layer is substituted, magnetization should increase with the substitution amount because it is the minority in the ferrimagnetic CoO_6_ layer. On the other hand, the substitution for the IS-state Co^3+^ in the ferrimagnetic CoO_6_ layer should decrease the magnetization. Hence, we suggest that Al^3+^ and Ga^3+^ are expected to substitute for the IS-state Co^3+^ in the CoO_6_ layer and HS-state Co^3+^ in the CoO_6_ or CoO_4.25_ layers, respectively. This suggestion is supported by the size of the ionic radius listed in Table 1. Al^3+^ is close to IS-state Co^3+^ in size, while Ga^3+^ is close to HS-state Co^3+^ in size.

Finally, we estimate *M*_s_ of Sr_3.1_Y_0.9_Co_4−*x*_*B_x_*O_10.5_ along with our suggestion. It is assumed that the LS-state Co ions induced by Al/Ga substitution distribute randomly. If Ga substitutes for the HS-state Co^3+^ in the CoO_6_ or CoO_4.25_ layers randomly, *M*_s_’s are estimated to be 0.271 and 0.292 μ_B_/Co at *x* = 0.2 and 0.4, respectively. Both values are in good agreement with the measured values shown in Figure 4b. On the other hand, if Al substitutes for the IS-state Co^3+^ in the CoO_6_ layer, *M*_s_’s are estimated to be 0.205 and 0.157 μ_B_/Co at *x* = 0.2 and 0.4, respectively. The values are somewhat smaller than the measured values. Therefore, Al mainly substitutes for the IS-state Co^3+^, but a part of Al would also substitute HS-state Co^3+^.

## 4. Conclusions

We measured and analyzed the magnetic properties of Co-substituted Sr_3.1_Y_0.9_Co_4−*x*_*B_x_*O_10.5_ (*B* = Al and Ga: *x* = 0, 0.2, and 0.4). We found that the Curie temperatures of both Sr_3.1_Y_0.9_Co_4−*x*_Al*_x_*O_10.5_ and Sr_3.1_Y_0.9_Co_4−*x*_Ga*_x_*O_10.5_ are about 1.5 times lower than the temperature estimated from the simple dilution effect. This extra Curie-temperature reduction is understood in terms of the LS-state Co^3+^ additionally induced by the Co site substitution. A mechanism of the LS-state Co^3+^ inducement by the substitution is not made clear in this study but should be revealed in future work. The saturation magnetization of Sr_3.1_Y_0.9_Co_4−*x*_Al*_x_*O_10.5_ decreases with Al content, whereas that of Sr_3.1_Y_0.9_Co_4−*x*_Ga*_x_*O_10.5_ increases with Ga content. This different behavior suggests the selectivity of the substitution site depends on the substituted element. Al^3+^ close to the IS-state Co^3+^ in ionic size suppresses the magnetization through substituting the majority component of the IS-state Co^3+^ in the ferrimagnetic CoO_6_ layer. By contrast, the substitution for the minority component of the HS-state Co^3+^ in CoO_6_ layer and/or HS-state Co^3+^ in antiferromagnetic CoO_4.25_ layer by Ga^3+^ enhances the magnetization. The present study suggests that the magnetization of the room-temperature ferromagnetic oxide Sr_3.1_Y_0.9_Co_4_O_10.5_ can be controlled by the selective substitution for Co site according to the ionic radius of the substituting elements.

## Figures and Tables

**Table 1:** Ionic radii of high-spin (HS), intermediate-spin (IS), and low-spin (LS) states Co^3+^, Al^3+^, and Ga^3+^.

Ion	Co^3+^(HS)	[33]	Co^3+^(IS)	[34]	Co^3+^(LS)	[33]	Al^3+^	[33]	Ga^3+^	[33]
Radius [Å]	0.61		0.56		0.545		0.535		0.62	
